# Robust Carbon Nanotube Transistor Ion Sensors with Near-Nernstian Sensitivity for Multi-Ion Detection in Neurological Diseases

**DOI:** 10.3390/nano15060447

**Published:** 2025-03-15

**Authors:** Lidan Yan, Yang Zhang, Zhibiao Zhu, Yuqi Liang, Mengmeng Xiao

**Affiliations:** 1 Hunan Institute of Advanced Sensing and Information Technology, Xiangtan University, Xiangtan 411105, China; 202221521383@smail.xtu.edu.cn (L.Y.); 202221521358@smail.xtu.edu.cn (Z.Z.); 2 School of Integrated Circuits, Beijing University of Posts and Telecommunications, Beijing 100876, China; zhangyang_ic@bupt.edu.cn; 3 Key Laboratory for the Physics and Chemistry of Nanodevices and Center, Carbon-Based Electronics School, Electronics Peking University, Beijing 100871, China; liangyuqi@stu.pku.edu.cn

**Keywords:** carbon nanotube, CNT FET, biosensors, ion sensors, ISFET, neurological disorders

## Abstract

Accurate monitoring of sodium and potassium ions in biological fluids is crucial for diseases related to electrolyte imbalance. Low-dimensional materials such as carbon nanotubes can be used to construct biochemical sensors based on high-performance field effect transistor (FET), but they face the problems of poor device consistency and difficulty in stable and reliable operation. In this work, we mass-produced carbon nanotube (CNT) floating-gate field-effect transistor devices with high uniformity and consistency through micro-/nanofabrication technology to improve the accuracy and reliability of detection without the need for statistical analysis based on machine learning. By introducing waterproof hafnium oxide gate dielectrics on the CNT FET channel, we not only effectively protect the channel area but also significantly improve the stability of the sensor. We have prepared array sensing technology based on CNT FET that can detect potassium, sodium, calcium, and hydrogen ions in artificial cerebrospinal fluid. The detection concentration range is 10 μM–100 mM and pH 3–pH 9, with a sensitivity close to the Nernst limit, and exhibits selective and long-term stable responses. This could help achieve early diagnosis and real-time monitoring of central nervous system diseases, highlighting the potential of this ion-sensing platform for highly sensitive and stable detection of various neurobiological markers.

## 1. Introduction

The normal function of neurons relies on the electrochemical balance between the intracellular and extracellular environments [1], particularly the concentrations of ions such as potassium (K^+^) [2], sodium (Na^+^), calcium (Ca^2+^), and chloride (Cl^−^) [3]. Abnormal fluctuations in the concentrations of these ions can lead to abnormal excitability or inhibitory effects in neurons, thereby affecting the conduction and processing of neural signals [4]. Numerous studies have reported correlations between the onset of neurological disorders and alterations in ion concentrations in cerebrospinal fluid (CSF) as shown in Table A1 [5]. The ability to selectively, sensitively, and simultaneously detect multiple ions in CSF is crucial for both monitoring the integrity of cortical function and aiding in the salvage of ischemic but potentially viable brain tissue [6].

Traditional ion detection methods, such as ion-selective electrodes [7], ion chromatography, and fluorescent probes [8], face limitations in sensitivity and complexity, prompting researchers to explore more efficient and integrable detection technologies. With the continuous advancement in microelectronics, FETs) have rapidly developed due to their small size, high sensitivity, and compatibility with complementary metal-oxide-semiconductor (CMOS) processes [9], offering high integration density. In particular, due to the large specific surface area of nanomaterials, FET sensors based on nanomaterials such as graphene [10] and carbon nanotubes [11] exhibit high sensitivity and fast response, allowing for real-time detection of ion concentration variations [12]. By employing an ion-selective membrane (ISM) as the sensitive layer, different concentrations of ions produce varying membrane potentials on the sensing layer, which in turn alters the threshold voltage and channel current of the nano-FET. Strategic integration of multiple ISM-modified FETs facilitates the multiplexed detection of diverse ions in cerebrospinal fluid, providing comprehensive neurochemical profiling for enhanced diagnostic precision. However, current nanomaterial-based FET sensors still face several challenges: (i) device-to-device variability and batch fabrication inconsistencies inherent to nanomaterial-based sensors; (ii) non-corrosion-resistant FET sensor structure under continuously applied voltages in highly ionic biofluids. Such non-uniformity and instability complicates calibration protocols and compromises detection reliability, ultimately hindering the commercial application of this technology [13].

Existing ion detection methods, in order to improve the accuracy of ion detection results, employ some machine learning approaches. These methods utilize a large number of devices for training and verification [14]. However, machine learning models require processing and analyzing large amounts of data, involving complex algorithms and computational processes, increasing the complexity of detection. Herein, we develop a mass-producible robust CNT floating-gate (FG) FET array with engineered uniformity and stability. The FG FET architecture synergistically enhances device consistency and protects the active channel from ionic interference. By improving the uniformity of carbon nanotube synthesis and device manufacturing technology, we reduce device-to-device variation, achieving mass production of CNT FET devices with high uniformity and consistency, without relying on machine learning for statistical analysis [15]. Combined with ISM modification, these CNT ion sensors achieve near-Nernstian sensitivities of 60 mV/dec for K^+^ and Na^+^, 39 mV/dec for Ca^2+^, and 71 mV/pH for H^+^ and accurate determination of the ion concentration without reliance on machine learning for statistical analysis. Moreover, we integrated the CNT FG FET ion sensors into a portable system, facilitating their application in home self-examination [16]. This work demonstrates that device-level collaborative optimization can also achieve highly reliable ion detection and reduce the burden on peripheral circuits and algorithms of the readout circuit system.

## 2. Materials and Methods

### 2.1. Fabrication of CNT FG FET

The fabrication method of the CNT FG FET sensor chip was the same as that previously reported by our laboratory [17]. By depositing polymer sorting solution-derived CNTs on Si/SiO_2_ substrates, randomly oriented CNT films with high uniformity and high semiconductor purity were obtained, and these were characterized by scanning electron microscopy (SEM). All preparation steps were patterned using photolithography, and Ti/Pd/Au (0.3 nm/40 nm/30 nm) sources and drain electrodes were formed by electron beam evaporation and lift-off techniques. The channel area was etched by oxygen plasma etching. A 3 nm thick hafnium film was deposited on the channel by atomic force deposition, and a 5 nm high-k hafnium oxide gate dielectric was formed by thermal oxidation in air for 30 min. Finally, the electrodes and leads were passivated with SU8 to protect them from the electrolyte. The optical image of the chip was characterized by optical microscopy, and the electrical properties of the chip were characterized by a probe station and a semiconductor parameter analyzer (Keithley 4200, Tektronix, Beaverton, OR, USA).

### 2.2. Reagents

Valinomycin, sodium ionophore X, calcium ionophore IV, poly(vinyl chloride), tetrahydrofuran, 2-nitrophenyl octyl ether and potassium tetrakis(4-chlorophenyl)borate were purchased from Aladdin (Hong Kong, China). Potassium chloride solution, sodium chloride solution and calcium chloride solution were purchased from Macklin (Germering, Germany). Sodium tetrakis [3,5-bis(trifluoromethyl)phenyl]borate bidepharm was purchased from Psaitong (Beijing, China). Bis(2-ethylhexyl)sebacate was purchased from Bide Pharmatech (Shanghai, China). Tris-HCl buffer solution (pH 7.4) was purchased from the official website of Biotech. ACSF (sterile) was purchased from Shanghai Yuanye Bio-Technology (Shanghai, China).

### 2.3. Preparation of ISMs

The potassium-selective membrane consisted of valinomycin (2% wt.%, ionophore), sodium tetrakis (4-chlorophenyl) borate (Na-TPB 0.5 wt.%, providing cation exchange centers and reducing resistance), poly (vinyl chloride) (32.7 wt.%, polymer matrix), and dioctyl sebacate (DOS 64.7 wt.%, plasticizer). The above mixture (100 mg) was dissolved in 660 µL of tetrahydrofuran (THF), forming a potassium ion-selective membrane solution.

The sodium-selective membrane was composed of sodium ionophore X (1 wt.%), sodium tetrakis [3,5-bis(trifluoromethyl) phenyl]borate (Na-TFPB, 0.55 wt.%), poly(vinyl chloride) (33 wt.%), and DOS (65.45 wt.%) and was prepared by dissolving 100 mg of the mixture in 660 µL of THF to yield the sodium ISM.

The calcium-selective membrane solution contained calcium ionophore IV (0.072%), Na-TFPB (0.022%), 2-nitrophenyl octyl ether (4.748%, plasticizer), poly (vinyl chloride) (2.379%), and tetrahydrofuran (92.78%, solvent). The prepared mixture was sonicated for 30 min to ensure complete dissolution, and the resulting membrane solution was stored at 4 °C in the dark.

The well-mixed membrane solution was sonicated for 30 min to ensure complete dissolution, and the prepared solution was stored at 4 °C in the dark. The channel area of the CNT FET was cleaned with deionized water and then dried with nitrogen gas. A pipette was used to dispense 5 μL of the prepared mixture onto the channel surface, followed by spin-coating at 600 rpm for 10 s and 2000 rpm for 60 s to evenly distribute the solution. The samples were left to dry overnight at room temperature, allowing the tetrahydrofuran to fully evaporate, thus forming various ISM-sensing interfaces for the gate electrodes of the potentiometric transistors. To enhance detection performance, a first layer of the ion-selective membrane was applied to the fabricated FET devices, and after the solvent had evaporated, a second layer of the ion-selective membrane was applied. The formation of a double-layer membrane can further reduce interference from other ions, improving overall selectivity and stability, and also enhances the sensor’s response to low-concentration ions. Before testing, the ion-selective membrane was activated with a 100 mM target solution for 1 h and then thoroughly rinsed with deionized water to restore the membrane potential to its initial level before commencing the test. The applied membrane must be stored in a moist environment because PVC membranes can crack when they become dehydrated, thereby affecting the sensing performance.

## 3. Results and Discussion

### 3.1. Fabrication and Characterization of CNT FG FET Ion Sensors

**Figure 1:**
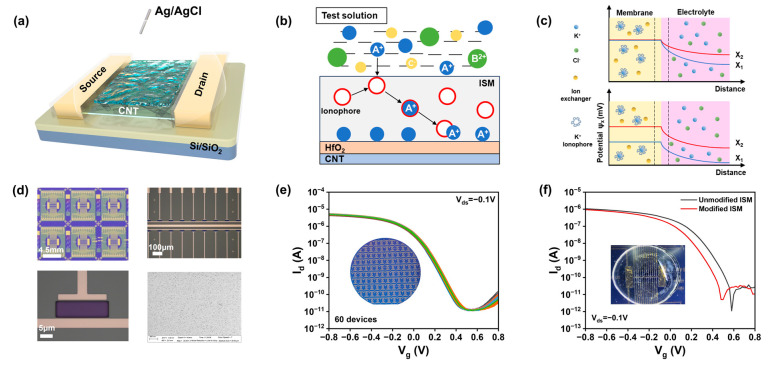
Structure and characteristics of a CNT FG FET ion sensor. (**a**) Structural diagram of a CNT FG FET ion sensor. (**b**) Schematic of the ISM selectively capturing ions. (**c**) Potential change when the sensor detects an ion. (**d**) Optical image of the manufactured CNT FET device. (**e**) Transfer curves of 60 devices at V_ds_ = −0.1 V. (**f**) Comparison of the device performance before and after modification of the ion-selective membrane.

**Figure A1:**
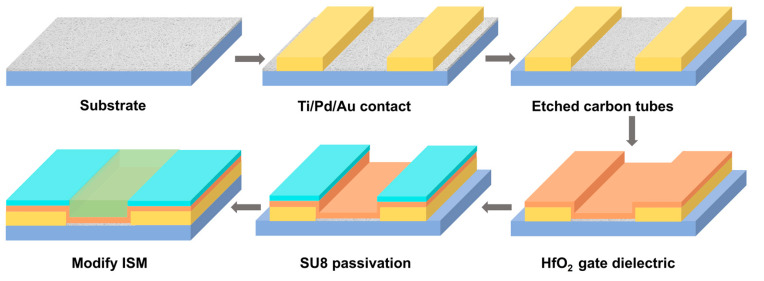
Device manufacturing process for CNT FG FET ion sensor.

One of the main challenges with silicon-based ion-sensitive field-effect transistors (ISFETs) is the corrosion of silicon and silicon oxide under high ionic strength. This corrosion alters the threshold voltage of the FET, leading to performance degradation over time [18]. In contrast, CNTs offer exceptional chemical and physical stability in physiological solutions due to their unique properties and structure. However, direct exposure of CNT channels to the environment can result in irreversible ion adsorption. This can cause local charge accumulation, negatively affecting the sensing performance and long-term stability of the device. To address this issue, we introduce a high-κ gate dielectric layer in our floating-gate structure with a hafnium oxide layer to enhance the ionic stability of the device. Figure 1a illustrates a schematic of the ISM-functionalized CNT FG FET ion sensor. The carbon-based sensing chip utilized in this study was fabricated on a 4-inch, 200 μm thick silicon wafer with carbon nanotubes. The CNT FG FET ion sensor was manufactured through a process depicted in Figure A1, which is analogous to previously reported methods, with all fabrication steps based on photolithography techniques. Each sensing unit consists of a 20 × 40 μm carbon nanotube channel and two Ti (0.3 nm)/Pd (40 nm)/Au (30 nm) source/drain electrodes. The CNT FG FET sensors were constructed in an array to enhance the reliability of sensor measurements. The optical image of the fabricated carbon-based field-effect transistor sensor is shown in Figure 1d, with each sensor chip incorporating 22 FET sensors.

To achieve reliable ion detection, the sensor must exhibit good uniformity and repeatability. We assessed the electrical performance of the fabricated ISFETs by measuring the transfer curves of 60 CNT FG FET devices at a source-drain voltage of −0.1 V in 100 mM Tris-HCl buffer solution (Figure 1e). The results indicate that the FETs possess highly uniform transfer characteristics, and the current variation coefficient of the device is within 7%. All transistors exhibit p-type FET behavior, with a highly uniform threshold, voltage transconductance, and subthreshold swing (Figure A3). The transfer characteristics curves reveal an excellent ion current on–off ratio (I_on_/I_OFF_) of 10^6^ and subthreshold swing (SS) of 87 mV/dec. The linear output curves at low biases (Figure A4) are attributed to ohmic contacts, which minimize contact resistance, mitigate the impact of channel resistance on the total device resistance, and ultimately maximize sensor response. Notably, the absence of hysteresis and the SS of 87 mV/dec suggest that, due to charge variations on the CNT FET, the CNT FET can provide stable and substantial changes in on-state current, which is crucial for the desired reliable detection. The gate leakage is maintained within 10 pA, which is negligible compared to the drain current over the entire range of gate bias operation.

### 3.2. Effect of Gate Dielectric on Ion Detection Performance

**Figure 2:**
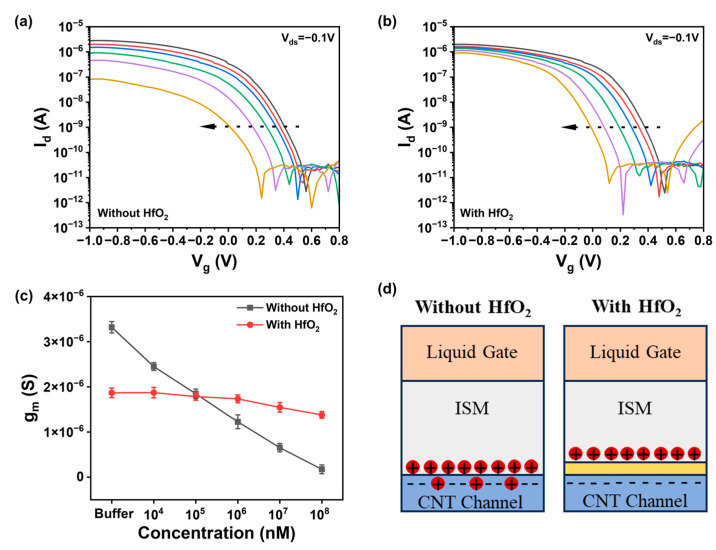
Effect of hafnium oxide gate media on ion detection. (**a**) Device performance tested multiple times when there is no hafnium oxide gate medium. (**b**) Device performance tested multiple times when there is hafnium oxide gate dielectric. (**c**) Transconductance changes in the extraction of with and without hafnium oxide devices. (**d**) Comparison of ion detection charge distribution with and without hafnium oxide.

In comparing the effects of the presence and absence of a gate dielectric on ion detection, we conducted multiple tests and obtained the transfer characteristic curve of the device as shown in Figure 2a,b. The direction of the arrow represents the increase in the number of tests. It was observed that the on-state current of the transfer characteristic curve of the device without a gate dielectric decreased significantly with multiple tests as shown in Figure 2a. The on-state current of the transfer characteristic curve of the device with a gate dielectric remained basically unchanged as shown in Figure 2b. Extracting the transconductance of the device from the transfer curve in Figure 2c, it can be seen that the transconductance of the device without a gate dielectric is constantly decreasing, while the transconductance of the device with a gate dielectric changes less, indicating that the stability of the device with a gate dielectric is better. This is because when there is no gate dielectric in the channel, a part of the ions enter the channel through the selective membrane, resulting in local charge accumulation and reduced stability of the device, as shown in Figure 2d [19]. When a gate dielectric is present, the gate dielectric layer prevents ions from entering the channel, thereby protecting the channel area from the influence of the solution environment and improving the long-term stability of the device. Therefore, even after multiple tests, the device with a gate dielectric still maintains good performance.

**Figure A2:**
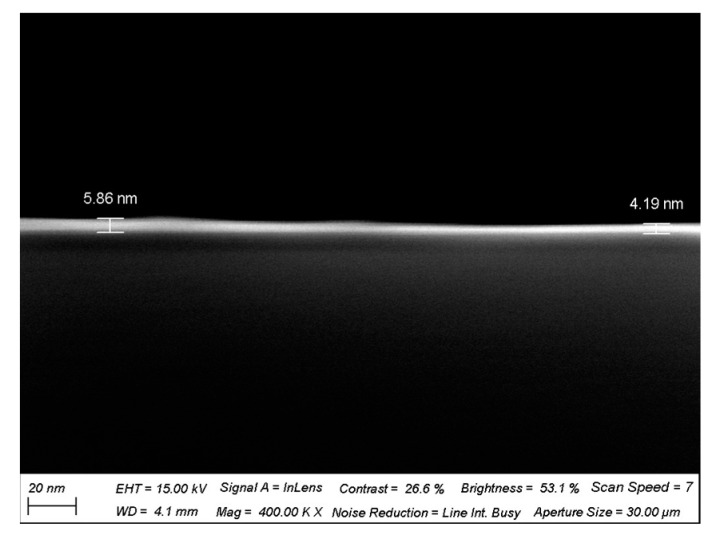
SEM cross-sectional image of hafnium oxide gate dielectric.

**Figure A3:**
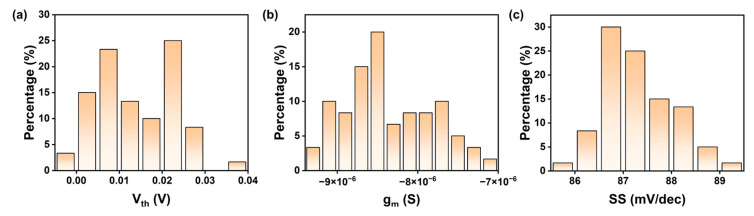
The electrical performance of the CNT FET devices. The statistics of (**a**) the threshold voltage, (**b**) the transconductance, and (**c**) the subthreshold swing.

**Figure A4:**
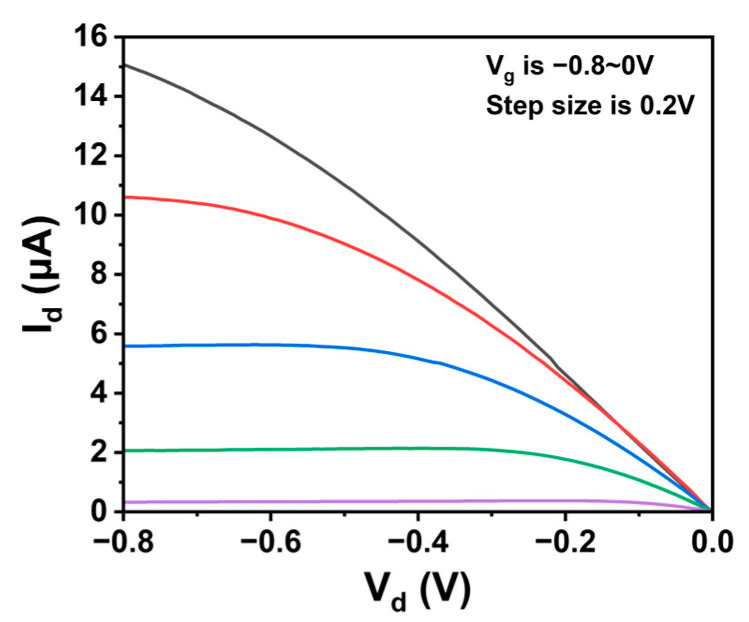
Output properties of a typical CNT FG FET.

Due to its high dielectric constant, hafnium oxide (HfO_2_) can provide greater gate control, thereby enhancing the sensitivity and switching characteristics of the device [20]. Hafnium oxide exhibits excellent resistance in various environments, which helps to improve the long-term stability and reliability of the device. Moreover, HfO_2_ is highly compatible with CNTs, facilitating the maintenance of CNT electronic properties and enabling integration with CNT FETs. To improve the stability of the ion sensor and maintain excellent detection performance in complex background environments, a 5 nm thick hafnium oxide high-k gate dielectric was deposited as a floating-gate insulating layer over the entire CNT channel, isolating the channel from the solution and enhancing the transistor’s gate control capability [21]. Figure A2 is an SEM cross-sectional view of the deposited HfO_2_ gate dielectric, which has a thickness of about 5 nm. A 500 nm SU8 passivation layer was spin-coated on top of the device to protect the electrodes and pads from the influence of the solution environment and to prevent device leakage, with patterning performed to leave openings in the sensing area above the carbon nanotube channel.

### 3.3. Detection Principle of CNT FG FET Ion Sensors

ISMs are polymeric membranes containing ionophore groups that possess the capability to selectively permeate ions within a solution [22]. By coating the surface of an ISFET with an ISM containing ionophores, the ISFET is endowed with selectivity towards the target ion. Figure 1b illustrates the process by which the ionophores within the ISM on the surface of the ion sensor bind with target ions in the solution; only ions that match the pore size and charge characteristics can pass through the membrane, while others are blocked. The interaction between the ionophores for K^+^, Na^+^, and Ca^2+^ and their corresponding target ions follows this mechanism [23], and the details of the ISM coating preparation are discussed in the experimental section. The performance of the ion sensor before and after the modification with the selective membrane was tested, and it was found that the carrier mobility of the CNT FET did not significantly change during the deposition of the ion membrane in Figure 1f. This indicates that the ion membrane performs a non-covalent functionalization of the carbon nanotubes, imparting ion sensitivity while maintaining carrier mobility.

**Figure A5:**
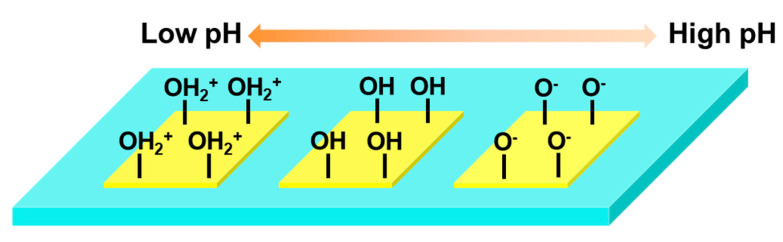
Principle of pH sensing.

The mechanism of pH sensing involves the reversible binding of hydrogen ions to the active hydroxyl groups on the surface of HfO_2_, as depicted in Figure A5. In acidic solutions, the hydroxyl groups (–OH) become protonated to form hydronium ions (–OH_2_^+^), leading to the accumulation of positive charges on the surface of HfO_2_. This causes the energy bands to bend further downwards, creating a higher hole barrier and ultimately reducing the on-state current (I_on_). Conversely, in an alkaline environment, the hydroxyl groups become deprotonated to form negatively charged oxygen atoms (–O^−^), resulting in the accumulation of negative charges on the surface of HfO_2_. This forms a lower hole Schottky barrier, thereby increasing the on-state current [24].

When the functionalized device comes into contact with an electrolyte solution, the ionophore within the ISM selectively captures the target ions in the solution, causing a change in the gate potential as shown in Figure 1c [25]. X_1_ is the initial ion concentration of the solution, and X_2_ is the ion concentration of the solution after the change. When the target ion concentration in the solution changes from X_1_ to X_2_, the concentration in the membrane remains in a buffer state and remains at a constant value. A charge separation layer (diffusion layer) with a thickness of several nanometers appears at the interface between the membrane and the solution. The concentration gradient between the solution and the membrane generates a potential difference on the diffusion layer. The potential at the electrolyte–membrane interface increases with the concentration of cations in the solution, necessitating an increased electric field to counteract diffusion, thus causing the potential to rise with increasing ion concentration [26]. An increase in cation concentration leads to greater p-doping of the channel, resulting in a leftward shift of the I-V characteristic curve. We can detect changes in the drain-source current to obtain information about the concentration of the target ions, which is the working principle of the ion-sensitive field-effect transistor. The relationship between the change in gate potential and ion concentration conforms to the Nernst equation, and the drain-source current is linearly related to the logarithm of ion concentration.

(1) $$\varphi_{i}=\varphi_{0}+2.303\frac{RT}{zF}\;lg\frac{M_{o}}{M_{i}}$$

In the equation, 2.303 is the conversion factor from the natural logarithm to the base-10 logarithm, *R* is the gas constant (8.135 J/(K·mol)), *T* is the absolute temperature in Kelvin, *z* is the ion valence, and *F* is Faraday’s constant (9.684 × 10^4^ C/mol). *M*_0_ and *M_i_* represent the ion concentrations outside and inside the membrane, respectively. Therefore, at room temperature, for monovalent ions (*z* = 1), the Nernst limit is 59 mV/decade. For divalent ions (*z* = 2), the Nernst limit is 29.5 mV/decade.

By characterizing the sensors with different ISMs, the versatility of the sensing system is demonstrated. When using neutral ionophores, it is necessary to incorporate lipophilic ion sites with the opposite charge of the analyte ion to prevent the extraction of chloride into the membrane. The concentration of these ion sites within the membrane can be optimized to effectively reduce response time, decrease membrane resistance, and enhance selectivity [27].

### 3.4. Performance of CNT FG FET Ion Sensors

All electrical tests were conducted using a probe station, with the Keithley 4200 semiconductor analyzer performing transfer curve and real-time tests on the FET sensors. To measure the *I_ds_*-*V_gs_* curves, the source-drain voltage (*V_ds_*) was set to −0.1 V, and the gate voltage (*V_gs_*) was scanned from −0.8 V to 0.6 V using an Ag/AgCl reference electrode, while simultaneously measuring the source-drain current (*I_ds_*). The Ag/AgCl reference electrode served as the electrolyte gate electrode to enhance the stability of the tests [28]. All experiments were carried out at room temperature in an electrolyte solution supported by 100 mM Tris-HCl buffer solution (pH 7.4), and all tests involving the electrical characteristics of the FET devices were performed with a liquid gate. Solutions of different concentrations were prepared using 100 mM Tris-HCl buffer solution to ensure consistent chloride ion concentration across the solutions, minimizing the impact of chloride ion concentration variations on the reference electrode potential and ensuring that the response was due to changes in ion concentration [29].

**Figure 3:**
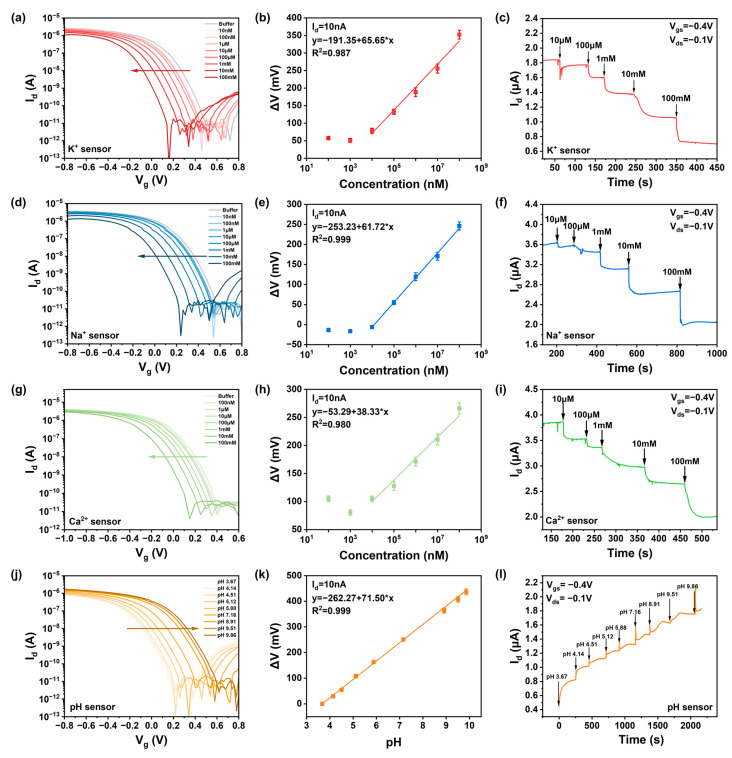
The response of different concentrations of ions was detected using an ISM-functionalized ion sensors. (The direction of the arrows represents the transition from low to high ion concentration and from low pH to high pH) (**a**) I_ds_-V_g_ curves of the K ion sensor for different concentrations of KCl solutions. (**d**) I_ds_-V_g_ curves of the Na ion sensor for different concentrations of NaCl solution. (**g**) I_ds_-V_g_ curves of the Ca ion sensor for different concentrations of CaCl_2_ solution. (**j**) I_ds_-V_g_ curve of the pH sensor for different pH solutions. Relationship between the concentration of ions extracted at 10 nA and value of voltage change: (**b**) K ion, (**e**) Na ion, (**h**) Ca ion, (**k**) pH. Dynamic response of ion sensor to different concentrations of target solution: (**c**) K ion, (**f**) Na ion, (**i**) Ca ion, (**l**) pH.

**Figure A6:**
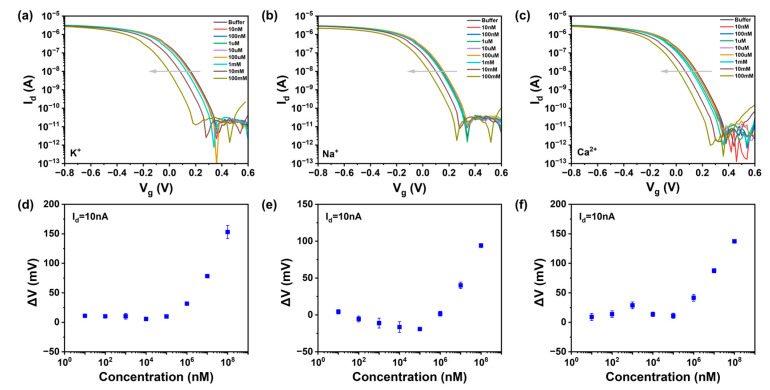
The response of the device to different concentrations of ions when the membrane is not homogeneous (The arrows mean the ion goes from low concentration to high concentration) (**a**,**d**) potassium, (**b**,**e**) sodium, (**c**,**f**) calcium.

This study demonstrated the versatility of the potentiometric sensor device by integrating CNT field-effect transistors with various ISMs, exhibiting high sensitivity and real-time response to the corresponding target ions (K^+^, Na^+^, Ca^2+^, and H^+^), as shown in Figure 3. The relationship between ion concentration and transfer characteristic curves in carbon-based field-effect transistors was investigated by measuring solutions of KCl, NaCl, CaCl_2_ within the range of 10 μM to 100 mM, as well as pH solutions from pH 3 to pH 9. When the device channel did not have an ion-selective membrane, the device responded minimally to ions within the concentration range in 100 mM Tris-HCl buffer solution [30], as seen in Figure A6, indicating that FET sensors without an ISM cannot distinguish the charge changes in different ions; hence, the bare chip cannot detect ion concentrations.

After testing the response of the background solution and waiting for stabilization, 7 μL of ion solution was pipetted onto the sensor channel; this was followed by electrical measurements. After measuring one concentration, the solution was blotted dry with a lint-free paper before the next concentration was applied. Figure 3 shows the response performance of sensors with ISM-functionalized gate electrodes in ion solutions within the concentration range of 10 μM–100 mM. As shown in Figure 3a,d,g, the increase in potassium, sodium, and calcium ion concentrations introduced more positive charges at the sensor–electrolyte interface, leading to a decrease in drain current in p-type devices and a regular leftward shift in the transfer curves. This indicates that ions in the solution can be effectively detected within the concentration range of 10 μM–100 mM. The pH sensor, as pH increases and H^+^ concentration decreases, shows an increase in drain current, causing the transfer curve to shift to the right as shown in Figure 3j. The threshold voltage is strongly logarithmically dependent on the molar concentration of specific ions. Based on transfer characteristic curve diagrams, the difference (Δ*V*) between the *V_i_* values corresponding to different concentration curves at *I_d_* = 10 nA and the *V_o_* of the background solution can be taken as a function of ion concentration, as shown in Figure 3b,e,h,k, yielding sensitivities of K-66 mV/dec, Na-62 mV/dec, Ca-39 mV/dec, and pH-71 mV/dec for the CNT FET ion sensors. The sensitivity of the CNT FET ion sensors approaches the Nernst limit of 59.6 mV/pH, achieving high sensitivity to potassium, sodium, calcium, and hydrogen ions over the concentration range of 10 μM–100 mM and pH 3 to pH 9.

By sequentially adding 2 μL of ion solutions ranging from 10 μM to 100 mM, the real-time current response of the ISM-functionalized CNT FET ion sensors was investigated. During testing, the source-drain bias (*V_ds_*) and gate voltage (*V_g_*) were set to −0.1 V and −0.4 V, respectively. When ions in the solution bind with the ionophores in the selective membrane, the gate potential of our p-doped device decreases, leading to a reduction in drain current. Figure 3c,f,i,l show the dynamic test results for the four types of sensors in response to solutions of different concentrations. The real-time drain-source current (*I_ds_*) is a function of ion concentration, and when solutions of different concentrations are added at different times, the device’s *I_d_* changes instantaneously, consistent with the trend of the static transfer curves. It was observed that the signal responds quickly to changes in ion concentration within 10 s, reaching a stable state after 50 s, indicating the FET biosensor’s characteristic of rapid response.

**Figure 4:**
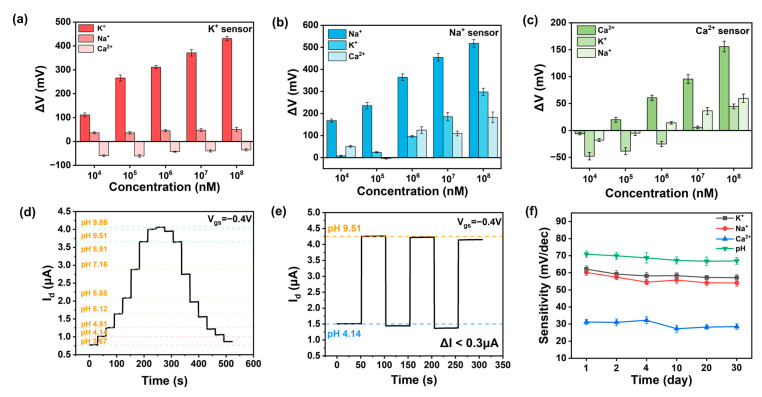
Selectivity and reversibility of CNT FG FET ion sensors. (**a**) Selectivity of the potassium ion sensor for sodium and calcium ions in the range of 10 μM–100 mM. (**b**) Selectivity of the sodium sensor for potassium and calcium in the range of 10 μM–100 mM. (**c**) Selectivity of the calcium sensor for potassium and sodium in the range of 10 μM–100 mM. (**d**) Real-time reversible current response of the pH sensor to 3.67–9.86 pH at a gate bias of −0.4 V. (**e**) Reproducibility of pH sensor between pH 4.14 and pH 9.51. (**f**) Stability of the ion sensors over a period of thirty days.

**Figure A7:**
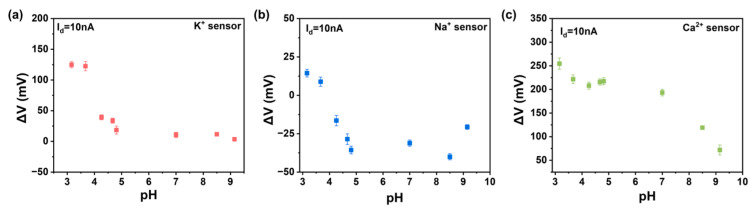
The response of ion sensors to pH: (**a**) potassium sensor, (**b**) sodium sensor, (**c**) calcium sensor.

Beyond sensitivity, selectivity is another critical requirement for high-performance ion sensors. We tested the response of various functionalized ion sensors to different concentrations of other ions, as shown in Figure 4a–c. It is evident that the response to the target ion is greater than that to other ions, thanks to the selective recognition by the ionophores in the ISM and the shielding effect of the double-layer membrane. Moreover, we tested the response of potassium and calcium ion sensors to different pH levels, as depicted in Figure A7a–c. It can be observed that the response is significantly smaller than that to the target ion, indicating that the pH level of the solution does not affect the results of ion detection. We define the selectivity of the sensor as the ratio of the concentration of non-specific ions required to achieve the same *V_ref_* value to the concentration of specific ions [31]. In the sodium-functionalized sensor, when KCl is at 10 μM, we obtained a current of 10 nA with a Δ*V* of 160 mV, corresponding to a sodium chloride concentration of 10 mM. The extracted selectivity of the sodium-functionalized sensor is 1000. The aforementioned research demonstrates that the sensor has a selective response to the target ion, confirming that the response of our sensor indeed originates from the specific binding between the ionophore and the target ion, rather than the ion strength. Comparing our work with other works, we found that our ion sensor has obvious high performance in Table 1. Our CNT FG FET is able to detect ions with high sensitivity and selectivity, mainly due to the synergistic effects of the ion-selective membrane, carbon nanotubes, and floating-gate structure.

For a reliable pH sensor, hysteresis and repeatability are also crucial. Repeatability refers to the variation in measurement results when the same measurement is repeated under identical conditions and in the same direction [32]. During testing, the source-drain bias (*V_ds_*) and gate voltage (*V_g_*) were set to −0.1 V and −0.4 V, respectively. We tested the dynamic response from pH 3.67 to 9.86 and back to 3.67, as shown in Figure 4d. The curves essentially coincide, indicating that the pH sensor has strong anti-hysteresis capabilities. We assessed the repeatability of the CNT FET ion sensor through cyclic dynamic testing at two different pH values (pH 4.14 and pH 9.15), as depicted in Figure 4e. The ISFET channel current showed almost no significant signal degradation (Δ*I* < 0.3 μA) over three cycles of concentration change, demonstrating the device’s good repeatability. These excellent performance characteristics once again confirm the favorable performance and minimally defective stable HfO_2_ sensing interface of the CNT FET ion sensor in continuous pH monitoring.

**Figure A8:**
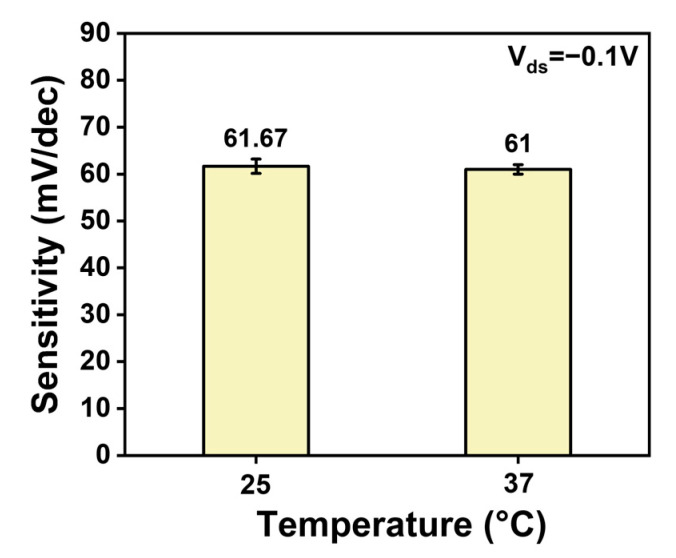
Comparison of ion sensor performance at 25 °C and 37 °C.

We further investigated the performance variation in the ISFET over time. Under the same bias voltage conditions, the stability of the ISFET was judged over thirty days by measuring the relationship between the response value (Δ*V*) and concentration. It can be seen that the sensitivity of ion sensors decreased slightly in the first four days and then remained essentially unchanged for the following month, with a variation of less than 10% in Figure 4f. In addition to the inherent stability of the carbon-based sensor, the outer layer of the membranes provide a stable ion shielding effect, protecting the ionophores in the inner layer from being washed away and enhancing the stability of the ion sensor [33]. The stability of the CNT FET ion sensors was satisfactory. We studied the effect of temperature on the sensing performance and found that the sensitivity did not change much when tested at 37 °C compared to 25 °C in Figure A8. The preservation of the membrane-functionalized device is also important. A lack of moisture can cause the membrane to crack, thereby affecting the performance of the ion sensor. Therefore, the devices were stored in 100 mM of the target solution at 25 °C.

### 3.5. Packaged CNT FG FET Ion Sensors for Portable Analysis of Artificial Cerebrospinal Fluid

**Figure 5:**
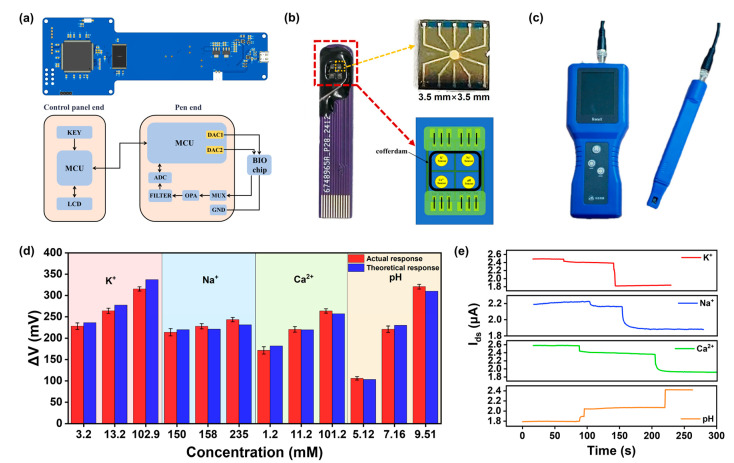
Detection of CNT FET ion sensors in artificial cerebrospinal fluid samples. (**a**) Circuit diagram and circuit design of the portable multi-ion detection system. (**b**) An optical picture of the multi-ion packaged chip and the multi-region packaged design. (**c**) An optical picture of the portable ion detector. (**d**) The actual response of ion concentration in artificial cerebrospinal fluid is compared with the theoretical response obtained from the fitting in Figure 3. (**e**) The simultaneous real time detection of K^+^, Na^+^, Ca^2+^, and pH in artificial cerebrospinal fluid.

Alterations in ion concentrations within the cerebrospinal fluid (CSF) are intimately associated with the onset and progression of various neurological disorders [5]. Monitoring these ion concentrations in the CSF can offer vital clues for the early diagnosis of neurological diseases. This study validated the potential applications of the sensor system’s for neurological disease monitoring by detecting K^+^, Na^+^, and Ca^2+^ in aCSF. To achieve portable ion detection, an ion tester was designed, composed of a custom printed circuit board (PCB) and a microcontroller, as shown in Figure 5a. The tester determines ion concentrations in solutions by measuring changes in potential across the sensors. The drain voltage (V_d_) of the ion chip is fixed at 1.65V, while the source voltage (V_s_) and gate voltage (V_g_) are not fixed. The control board MCU can control the DAC1/DAC2 outputs of the acquisition board MCU to provide V_s_/V_g_. When the MCU outputs V_s_/V_g_ to the source/gate of the ion chip through the internal DAC1/DAC2, the ion chip responds with nA-level currents through eight drain outputs. The multiplexer (MUX) system sequentially selects each drain channel current to the input of the operational amplifier (OPA). The nA-level response current is amplified to the mV-level voltage by the OPA and fed into the ADC input for the MCU to acquire. The MCU calculates the response current and transmits the value through the TTL serial port to the control board MCU. The control board MCU then fits the relationship between the response current and ion concentration and tests the ion concentration of the solution to be measured, displaying the ion concentration value on the LCD.

In this study, we developed a multi-region packaged CNT FET chip for the simultaneous detection of multiple ions using an ion detector. The simultaneous detection of multiple markers is very important for achieving comprehensive disease analysis. However, due to the limitations of traditional drip-coating functionalization methods, multi-modified chips often require a large chip size to isolate each region, which greatly reduces the number of chips integrated on a wafer and increases chip costs [34]. To achieve higher integration, wire bonding and epoxy damming techniques were employed to package the CNT FET chip, similar with our previous study [35]. Wire bonding was used to establish reliable electrical connections between the chip and external circuits, and epoxy damming was used to protect the metal wires and electrodes from liquid environments and mechanical vibrations. The packaged CNT FET chip demonstrated high uniformity and stability, with multiple channels exhibiting high consistency and linear response during the detection process. In our multi-region packaged chip design, the four small-sized CNT FG FET ion-sensing chips (with dimensions of 3.5 mm × 3.5 mm) were placed in the four regions of the PCB base, which were isolated from each other using the epoxy damming technique. Each region can be functionalized with a different ion-selective membrane separately, enabling the simultaneous detection of four distinct ions, as shown in Figure 5b. This design allows for the use of shared biological samples for concurrent testing without the need for complex microfluidic assistance. This package process also is compatible with functionalization with different biomolecules.

Combining the ion detection system and the multi-ion packaging chip will form a portable multi-ion detector, as shown in Figure 5c. We used the portable detector to test the concentrations of four ions in artificial cerebrospinal fluid. As seen in Figure 3, we input the calibration curve of each ion into the internal program of the multi-ion detector. We compared the detection values of different ion concentrations in cerebrospinal fluid with theoretical values, as shown in Figure 5d. It can be seen that in the presence of other molecules and ions, the measured ion concentrations differ from the actual concentrations by less than 5%, proving that our sensors can achieve highly sensitive, selective, and stable detection in complex samples. By combining the multi-ion package chip and the ion detector, the concentration of four ions in the solution can be detected simultaneously. The concentrations of K^+^, Na^+^, and Ca^2+^ were 333.33 µM, 3.33 mM, and 33.33 mM, respectively, while the pH values tested were 4, 7, and 9. As the ion concentrations increased, the drain current of the CNT FET chip decreased significantly, demonstrating the excellent performance of the multi-ion packaged chip, as shown in Figure 5e. Our portable ion detector provides a rapid, accurate, and cost-effective method for detecting multiple ion concentrations, meeting the needs of on-site, rapid ion detection.

## 4. Conclusions

We have successfully designed a CNT FET device, capitalizing on its exceptional uniformity and reproducibility to fabricate an array capable of concurrently detecting potassium (K^+^), sodium (Na^+^), calcium (Ca^2+^), and hydrogen (H^+^) ions. The incorporation of a hafnium oxide gate dielectric within the channel significantly enhances the stability of ion sensing. The CNT FG FET devices, functionalized with ISMs, exhibit superior sensitivity, selectivity, and stability in ion detection, achieving detection within the range of 100 μM to 100 mM under 100 mM Tris-HCl buffer solution conditions, with sensitivity approaching the Nernstian limit. Additionally, by encapsulating the ion-sensing chips and designing a portable ion detection system, we have realized multi-channel ion detection in cerebrospinal fluid. The sensor maintains consistent performance over a month, demonstrating excellent reusability and offering a novel approach for the diagnosis and prevention of neurological diseases.

## Figures and Tables

**Table 1:** Comparison of this work with other works.

Target	Device	Range	Sensitivity	Selectivity	Reference
Na^+^	Organic Silicon Elastic Body ISE	1 mM–1 M	56.1 mV/dec	K = −3.0	[7]
K^+^	Graphene FET	10 nM–1 mM	7.8 mV/dec	Na = −0.18 mV/dec	[29]
K^+^, Na^+^	Graphene FET Array	1 μM–50 mM	45.7 mV/dec, 49.2 mV/dec		[23]
K^+^	Graphene FET with Xylene Encapsulation	1 nM–100 mM	37 mV/dec	Na = 2.5 mV/dec, Ca = 4.2 mV/dec	[26]
K^+^, Na^+^, Ca^2+^	Graphene Electrolyte Gate Field-Effect Transistor	10 μM–100 mM	54.7 mV/dec, 56.8 mV/dec, 30.1 mV/dec		[22]
K^+^, Ca^2+^	SiNW-FET	10 mM–100 mM	43.33 mV/dec, 29.47 mV/dec		[5]
K^+^, Na^+^, Ca^2+^, pH	CNT FET	10 μM–100 mM pH 3–pH 9	65 mV/dec 61 mV/dec 38 mV/dec 71 mV/pH	Log K= 1000 Log Na = 1000 Log Ca = 100	This Work

## Data Availability

The original contributions presented in the study are included in the article, further inquiries can be directed to the corresponding author.

## Appendix A

**Table A1:** Relationship between neurological disorders and changes in ion concentrations.

Neurological Disorder	K^+^	Na^+^	Ca^2+^	H^+^	Symptoms
Epilepsy	✓	✓	✓		Instant loss of consciousness and falls
Traumatic brain injury	↑	↓			Brain dysfunction
Cerebral ischemia				↑	Acidosis
Hyponatremia		↓			Dizziness or coma
Hyperkalemia	↑				Muscle spasms
Multiple sclerosis (MS)	✓		✓		Limb weakness, paresthesia
